# Dr. Samuel Fothergill

**Published:** 1822-12

**Authors:** 


### OBITUARY.

Lately, in the Island of Jamaica, aged 44, Dr. SAMUEL FOTHERGILL, for many years a physician of eminence in London, and one of the able conductors of this Journal. Dr. Fothergill was born in Yorkshire, and, after having received the rudiments of his professional education, repaired to Edinburgh, where he graduated, and came to London. He was soon elected physician to the Westminster General Dispensary, the duties of which office he zealously performed for many years; but his health being impaired by residence in the metropolis, and having suffered several attacks of hæmoptysis, he determined to relinquish his prospects in London, and to seek the restoration of his health by a change of climate.

He practised as a physician, with distinguished success, in Jamaica for some years, but was interrupted several times by recurrence of hemorrhage from the lungs; to which, and the debility it occasioned, he at last fell a sacrifice.

Dr. Fothergill possessed many amiable qualities of heart, which endeared him to a number of friends, and professional talents and erudition, which, if his health had permitted, would have led him to great distinction among his professional brethren in London, by whom he was highly respected.

*(Our account of* Dr. MARCET *is unavoidably postponed.)*

---

☞ *The number of Original Communications transmitted to us has been so great, that, besides increasing the portion of the Journal usually assigned for their insertion, we have been induced to place some of the shorter papers at the end, under the department of "Intelligence," which, it will be observed, is in this Number entirely original.*

